# The Value of PD-L1 Expression in Predicting the Efficacy of Anti-PD-1 or Anti-PD-L1 Therapy in Patients with Cancer: A Systematic Review and Meta-Analysis

**DOI:** 10.1155/2020/6717912

**Published:** 2020-12-16

**Authors:** Xiao-Jiang Chen, Shu-Qiang Yuan, Jin-Ling Duan, Yong-Ming Chen, Shi Chen, Yun Wang, Yuan-Fang Li

**Affiliations:** ^1^Department of Gastric Surgery, Sun Yat-sen University Cancer Center; State Key Laboratory of Oncology in South China; Collaborative Innovation Center for Cancer Medicine, Guangzhou, China; ^2^State Key Laboratory of Oncology in South China, Collaborative Innovation Center for Cancer Medicine, Guangzhou, China; ^3^Department of Gastric Surgery, The 6th Affiliated Hospital, Sun Yat-sen University, Guangzhou, China; ^4^Department of Hematological Oncology, Sun Yat-sen University Cancer Center; State Key Laboratory of Oncology in South China; Collaborative Innovation Center for Cancer Medicine, Guangzhou, China

## Abstract

**Objectives:**

Recent trials have shown an overall survival (OS) benefit in 10-40% advanced cancer patients treated with programmed cell death 1 (PD-1) or programmed death-ligand 1 (PD-L1) inhibitors. Here, we aimed to evaluate the relationship between PD-L1 expression and the therapeutic efficacy of PD-1 or PD-L1 inhibitors in patients with cancer with recurrent or metastatic disease, compared with control treatments.

**Methods:**

We systematically searched Medline (PubMed), Embase, and Cochrane Library databases up to Jan 2019 and pooled the treatment effects (hazard ratio or relative ratio) of PD-1/PD-L1 inhibitors in patients with different PD-L1 expression.

**Results:**

Overall, twenty-four qualifying trials with over 14,860 subjects were eligible in this study. Compared with conventional agents, anti-PD/PD-L1 drugs significantly reduced the risk of death (hazard ratio 0.72, 95% CI 0.66 to 0.78), irrespective of the tumor type. Additionally, when PD-L1 expression ≥1% was defined as positive, anti-PD-1/PD-L1 monotherapy correlated with prolonged overall survival in patients with nonsmall cell lung cancer (NSCLC) (0.72, 0.61 to 0.86) and other cancer types (0.66, 0.57 to 0.76) patients with PD-L1 positive, rather than those with PD-L1 negative (hazard ratio for NSCLC and other cancer types: 0.84 and 0.87, respectively; all *P* > 0.05). The subgroup analyses to experimental agents, PD-1/PD-L1 inhibitors, PD-L1 antibody clone, and type of IHC scoring method validated the robustness of these findings. However, anti-PD-1/PD-L1 combination therapies can reduce the risk of death for patients with different cancer types, regardless of PD-L1 expression (*P* < 0.05 for all PD-L1 expression status).

**Conclusions:**

We recommend PD-L1 expression as a predictive biomarker in patient selection for anti-PD-1/PD-L1 monotherapy, but not for combination therapies.

## 1. Introduction

Over the recent decades, the therapeutic blockade of immune checkpoints has led to one of the most unprecedented breakthroughs in cancer treatment, especially for advanced, metastatic, or recurrent solid tumors [1, 2]. The immune checkpoint inhibitors fight against cancers by enhancing antitumor immunity based on a series of coinhibitory and costimulatory receptors and their ligands, known as immune checkpoints. In 2011, the United States Food and Drug Administration (FDA) approved the first immune checkpoint inhibitor, ipilimumab, a monoclonal antibody targeting cytotoxic T lymphocyte-associated antigen 4 (CTLA-4), for the treatment of metastatic melanoma. However, the more promising immunotherapy is now programmed death 1 (PD-1) or PD ligand 1 (PD-L1) inhibitors, since the PD1/PD-L1 axis plays a key role in physiological immune homoeostasis and tumor immune evasion. The development and application of antibodies targeting PD-1 (nivolumab and pembrolizumab) and PD-L1 (atezolizumab, avelumab, and durvalumab) have yielded favourable therapeutic effects on various solid tumors, such as lung cancer, melanoma, renal cell carcinoma, Hodgkin's lymphoma, urothelial cancer, head and neck cancer, gastric cancer, Merkel cell carcinoma, and mismatch repair deficiency or microsatellite instable-high solid tumors [3–6]. At present, PD-1 and PD-L1 inhibitors have been licensed to treat a variety of cancers and are being investigated in more than 1000 clinical trials.

Although PD-1/PD-L1 inhibitors exerted a revolutionary effect on cancer treatment, there are several critical issues restricting the extensive clinical utility of PD-1/PD-L1 inhibitors. For one, only a minority of patients (varying from 10% to 40%) exhibited durable antitumor responses and favourable long-term outcomes after receiving PD-1/PD-L1 inhibitors, and intrinsic drug resistance is common [7]. Additionally, immunotherapy is associated with several immune-related adverse events and can be very costly. Therefore, there is a critical issue under investigation [7]: how to predict the response and survival outcome before the initial therapeutic use of a PD-1-PD-L1 blockade. Currently, several biomarkers, such as PD-L1 expression, tumor mutation burden [8], virus infection [9], and genetic mutations within cancer cells [10], have been investigated to determine if they are associated with the treatment efficacy of PD-1/PD-L1 inhibitors. Among these biomarkers, overexpressing PD-L1 expression on the one side is associated with worse prognosis in cancer patients [11] and on the other hand is considered a biologically plausible and targetable available biomarker in predicting the tumor response and survival prognosis. Several randomized controlled trials [12] have shown a higher overall response rate to PD-1/PD-L1 inhibitors and prolonged overall survival in patients who are PD-L1 positive, rather than those who are PD-L1 negative. Considering the mechanism of the PD-1/PD-L1 inhibitor treatment, it seems logical that PD-L1 expression should be correlated with clinical outcomes. However, the proposition to use PD-L1 expression status for predicting anti-PD-1/PD-L1 immunotherapy is challenging for several reasons; this includes the reporting of different cut-offs for definitions of positive and negative expression, which is further compounded by the possibility of interlaboratory variation. Therefore, it is essential to perform a pooled analysis to clarify the utility of PD-L1 expression in predicting the efficacy of anti-PD-1/PD-L1 immunotherapy.

Based on this premise, Shen et al. [13] performed a meta-analysis to evaluate the efficacy of PD-1 or PD-L1 inhibitors in patients with cancer that were PD-L1 positive and PD-L1 negative; they concluded that PD-L1 expression could not sufficiently predict the therapeutic efficacy of PD-1/PD-L1 inhibitors. However, they pooled the results through only a small number of trials (8 randomized trials) of various cancer types, which could confound and bias their results. Additionally, they set a cut-off value of >1% as the definition of PD-L1-positive expression, and it remains uncertain whether 1% was the optimal cut-off value. Here, with the accumulated evidence, we conduct a systematic review and meta-analysis to evaluate the relationship between PD-L1 expression and the efficacy of anti-PD-1/PD-L1 therapy.

## 2. Methods

We conducted this study in adherence with the Cochrane Handbook for Systematic Reviews of Interventions and reported our findings based on the Preferred Reporting Items for Systematic Reviews and Meta-Analyses (PRISMA) guidelines [14].

### 2.1. Search Strategy and Selection Criteria

Two authors (XJC and JLD) electronically searched the Medline (PubMed), Embase, ClinicalTrials.gov, and Cochrane Library databases to identify randomized controlled trials that compared anti-PD-1/PD-L1 drugs to control drugs for solid tumors from inception up to Jan 2019 with no restriction on language. We used a manual search strategy to review references of the included trials and abstracts of two conference proceedings (2019 American Society of Clinical Oncology [ASCO] annual meeting and the European Society for Medical Oncology [ESMO] 2018 congress) to retrieve additional studies. We searched for the following concepts and linked them together with the OR/AND operator: nivolumab, pembrolizumab, avelumab, atezolizumab, durvalumab, PD-1, PD-L1, checkpoint inhibitors, and randomized controlled trial [15]. The complete list of search terms is shown in Box 1 (supplementary materials).

The purpose of this study was to evaluate the relationship between PD-L1 expression and the efficacy of anti-PD-1/PD-L1 therapy and identify the potential cut-off value for PD-L1 expression to distinguish the efficacy of anti-PD-1/PD-L1 therapy. We therefore prespecified the inclusion criteria according to population-intervention-comparison-outcome (PICO) guidelines. Foremost, for the participant: the PD-L1 expression status of the included participants (aged >18 years) was assessed; PD-L1 expression was defined as the percentage of tumor cells or tumor and immune cells that were PD-L1 stained by immunohistochemistry (IHC) methods. Next, for the intervention: treatment with anti-PD-1/PD-L1 drugs irrespective of monotherapy or combinational therapy. Then, for the comparison: conventional treatment without anti-PD-1/PD-L1 drugs. Lastly, for the outcome: primary outcome was overall survival calculated as hazard ratio (HR) stratified by PD-L1 expression status.

We excluded reviews, commentaries, retrospective studies, phase 1 or nonrandomized phase 2 studies, studies not published as full-text articles, quality of life studies, cost effectiveness analyses, studies in which the effect of the drug could not be ascertained, such as when the control was a different dose of the same drug, and studies without a report of overall survival by PD-L1 expression.

### 2.2. Data Extraction and Quality Assessment

Two authors (XJC and SQY) extracted the following characteristics for each trial: phase of study (phase 2 or 3), included population, line of therapy, treatment regimen, type of PD-L1 antibody clone, PD-L1 IHC scoring method, number of the patients by PD-L1 expression, median follow-up time, and HR for overall survival and relative risk (RR) for objective response rate according to different PD-L1 expression (Table 1). Two authors (SQY and SC) used the Cochrane Risk of Bias Tool to evaluate the risk of bias in all the included trials in this review [16]. Discrepancies in the literature search and data extraction were resolved by two independent authors (ZWZ and YFL). Differences were resolved by consensus.

### 2.3. Data Synthesis and Analysis

The primary endpoint of our study was the overall survival, defined as the time from randomization to death from any cause. The secondary endpoint was objective response rate, measured as the proportion of confirmed complete response or partial response at the best response. Tumor responses were assessed by the Response Evaluation Criteria in Solid Tumours (RECIST) version 1.1 criteria [17]. Accordingly, we calculated the HR and 95% confidence intervals (CI) for overall survival and the RR and 95% CI for objective response rate in each study separately in patients with different PD-L1 expression.

We used the *I*^2^ index and the Cochran Q statistic to assess the heterogeneity between different trials, with *I*^2^ > 50% and *P* < 0.1 indicating significant heterogeneity [18]. If the trial was considered homogeneous (*P* > 0.1), pooled treatment effects (HR/RR and 95% CI) were calculated through a fixed effects model using the inverse variance method; otherwise, a random effects model was preferred. For studies reporting more than one intervention arm (CheckMate 067 [19]: nivolumab plus ipilimumab arm and nivolumab arm), we compared the intervention arms with the control arm separately. We performed subgroup analyses to evaluate studies by different tumor types (nonsmall cell lung cancer (NSCLC) and other cancer types) and treatment strategies (monotherapies and combination therapies) in different PD-L1 expression status. Additionally, subgroup analyses stratified by experimental agents, PD-1/PD-L1 inhibitors, PD-L1 antibody clone, and type of IHC scoring method were conducted. An interaction test was used to evaluate the heterogeneity of efficacy between subgroups, expressed as P for interaction. We assessed the potential publication bias by funnel plots and evaluated the data through the Egger and Begg tests, with *P* < 0.1 considered significant [20, 21]. All statistical analyses were performed using Stata version 12.0 (StataCorp, College Station, TX). Two-sided *P* < 0.05 was considered statistically significant.

## 3. Results

Figure S1 shows that we identified 486 related studies after the initial search strategy; 436 studies were further excluded after screening the title and abstract. After review of the full-text of 50 articles, 23 studies meet the inclusion criteria. We then performed a careful manual search and identified one study for inclusion. Hence, a total of 24 trials were included for the quantitative synthesis and meta-analysis [19, 22–44]. All the included studies were published between January 2015 and October 2018. The funnel plot and Begg rank correlation test (*P* = 0.137) indicated no obvious publication bias (Figure S2).

### 3.1. Study Characteristics

Table 1 shows the detailed information of the eligible studies. All the included trials were international multicentre studies funded by the pharmaceutical industry, including 2 phase 2 trials, 1 phase 2/3 trial, and 21 phase 3 trials, with the sample sizes ranging from 142 to 945 subjects. The median follow-up time of the included trials varied from 5.1 months to 38 months. All the 24 trials were conducted in metastatic or recurrent settings, among which twelve were performed in NSCLC [22, 23, 26, 28, 30, 32, 34, 36, 40–42, 44], five in melanoma [19, 25, 29, 35, 37], two in renal cell carcinoma [24, 38], two in gastric or gastroesophageal junction cancer [33, 39], one each in head and neck cancer [27], urothelial carcinoma [31], and triple-negative breast cancer [43]. Patients in the experimental arm received nivolumab in 11 trials [19, 22–25, 27, 29, 32, 33, 37, 38], pembrolizumab in 7 trials [28, 30, 31, 35, 36, 39, 42], atezolizumab in 4 trials [26, 34, 43, 44], and avelumab [41] and durvalumab [40] in 1 trial each. Six trials evaluated the combination of anti-PD-1/PD-L1 drugs with ipilimumab [29, 38] and chemotherapy [36, 42–44] compared with conventional treatment; seventeen trials assessed the efficacy of anti-PD-1/PD-L1 monotherapies [22–28, 30–35, 37, 39–41]; one trial, namely, CheckMate 067 [19], was purposed to evaluate the therapeutic effect of nivolumab monotherapy or combination therapy with nivolumab plus ipilimumab in melanoma compared with ipilimumab. The threshold of PD-L1-positive expression was defined as 1% in eighteen trials [19, 22–24, 26–28, 33–36, 38–44], 5% in four trials [25, 29, 32, 37], and 10% [31] and 50% [30] in one trial each.

The risk of bias assessments for the included trials is shown in Table S1. All studies but one generated the randomized treatment allocation sequences. The quality of the included trials was considered moderate to good; the main factor impacting the quality was the open-label design of most of the included trials.

### 3.2. Therapeutic Efficacy of Anti-PD-1/PD-L1 Drugs

The 24 eligible trials to evaluate the therapeutic efficacy of anti-PD-1/PD-L1 drugs comprised a total of 14,860 patients, with 8212 subjects in the intervention arms (nivolumab: 3310, pembrolizumab: 2654, atezolizumab: 1376, avelumab: 396, and durvalumab: 476) and 6648 subjects in the control arms (chemotherapy: 4459, ipilimumab: 955, targeted drug: 833, and placebo: 401).

**Figure 1:**
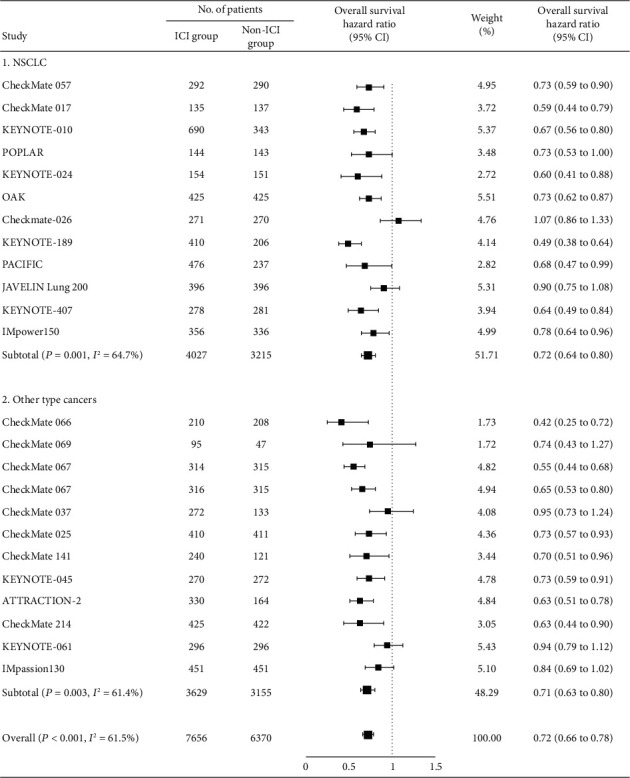
Forest plot of overall survival in patients treated with anti-PD-1/PD-L1 drugs versus control. PD-1: programmed death 1; PD-L1: programmed death-ligand 1.

We observed significant heterogeneity (*I*^2^ = 61.5%, *P* < 0.001) among the eligible studies. A random effects model with an inverse variance method was therefore preferred in the present meta-analysis. Overall, we noted that anti-PD/PD-L1 drugs increased tumor response (RR 1.91, 95% CI 1.62-2.26, *P* < 0.001; Figure S3) and reduced the risk of death significantly (HR 0.72, 95% CI 0.66-0.78, *P* < 0.001; Figure 1), irrespective of tumor type, compared with the control treatment.

### 3.3. PD-L1 Expression to Predict the Therapeutic Efficacy

Next, we examined the relationship between PD-L1 expression and the efficacy of anti-PD/PD-L1 immunotherapy. Given that there may be heterogeneity between trials, we performed subgroup analyses stratified by different tumor types (NSCLC and other cancer types) and treatment strategies (monotherapies and combination therapies).

**Figure 2:**
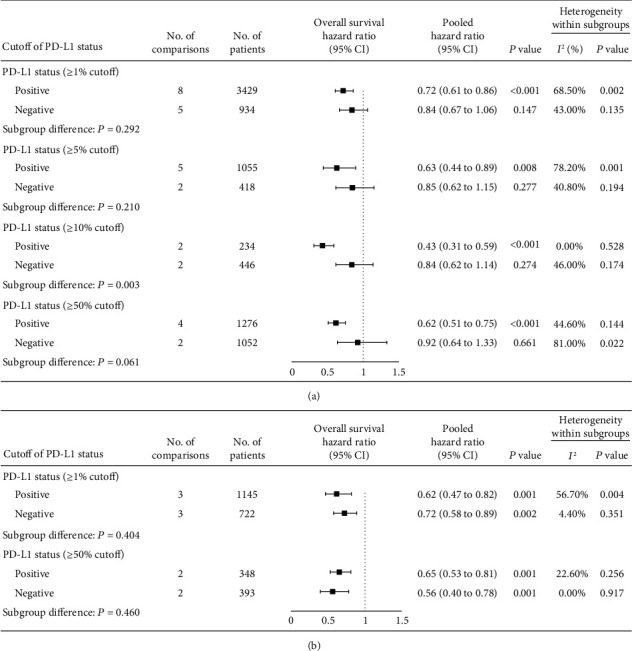
(Forest plot of overall survival comparing anti-PD-1/PD-L1 drugs to control treatments in NSCLC patients with different PD-L1 expression statuses. NSCLC: nonsmall cell lung cancer; PD-1: programmed death 1; PD-L1: programmed death-ligand 1.

For NSCLC patients, anti-PD-1/PD-L1 monotherapy was superior to control treatment in terms of objective response rate (RR_1%_ 1.61 [1.33-1.95] vs. RR_5%_ 2.12 [1.49-3.00] vs. RR_10%_ 1.76 [1.11-2.81] vs. RR_50%_ 2.35 [1.41-3.91]; all *P* < 0.05, Figure S4A) and overall survival (HR_1%_ 0.72 [95% CI 0.61-0.86] vs. HR_5%_ 0.63 [0.44-0.89] vs. HR_10%_ 0.43 [0.31-0.59] vs. HR_50%_ 0.62 [0.51-0.75]; all *P* < 0.01, Figure 2(a)) in patients who were PD-L1 positive, but not in those who were PD-L1 negative (objective response rate RR_1%_ 0.84 vs. RR_5%_ 0.84 vs. RR_10%_ 1.01 vs. RR_50%_ 0.96; all *P* > 0.05, Figure S4A; overall survival HR_1%_ 0.84 vs. HR_5%_ 0.85 vs. HR_10%_ 0.84 vs. HR_50%_ 0.92; all *P* > 0.05, Figure 2(a)). Moreover, we noted a dose-response relation with a higher cut-off improving survival in PD-L1 positive NSCLC patients (HR_1%_ 0.72 vs. HR_5%_ 0.63 vs. HR_10%_ 0.43vs. HR_50%_ 0.62; interaction test *P* = 0.050, Figure 2(a)). In terms of objective response rate, anti-PD-1/PD-L1 antibody in combination with other conventional drug increased tumor response in patients who were PD-L1 positive (RR_1%_ 1.83 vs. RR_50%_ 2.17; all *P* < 0.05, Figure S4B) rather than those who were PD-L1 negative (RR_1%_ 1.41 vs. RR_50%_ 1.64; all *P* > 0.05, Figure S4B); nonetheless, these combination therapy was associated with prolonged overall survival regardless of PD-L1 expression (HR for PD-L1 <1% vs. ≥1% [0.72 vs. 0.62], <50% vs. ≥50% [0.56 vs. 0.62]; all *P* < 0.01, Figure 2(b)).

**Figure 3:**
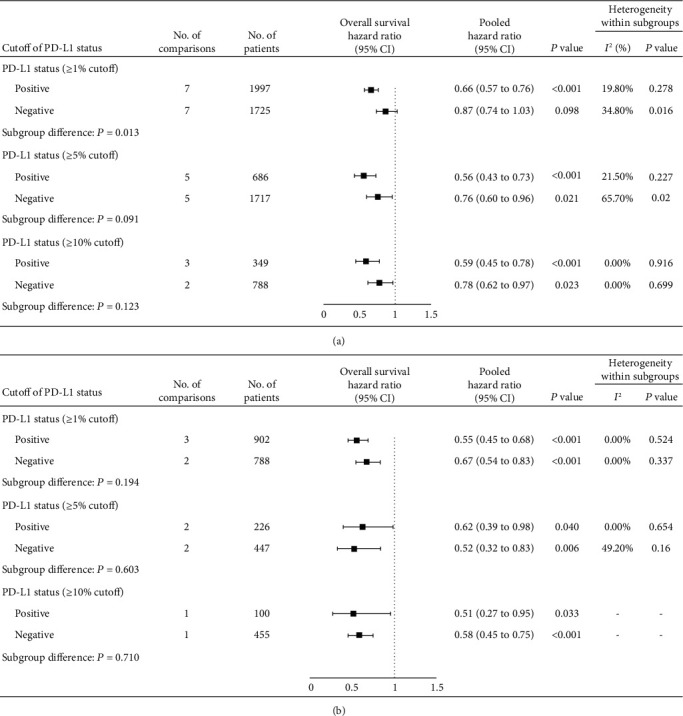
Forest plot of overall survival comparing anti-PD-1/PD-L1 drugs to control treatments in patients with other cancer types with different PD-L1 expression statuses. PD-1: programmed death 1; PD-L1: programmed death-ligand 1.

For patients with other cancer types, anti-PD-1/PD-L1 monotherapy was associated with increased tumor response in patients with PD-L1 expression ≥1% (RR 2.62 [1.47-4.68]; *P* = 0.001, Figure S5A) but not in those with PD-L1 expression <1% (RR 0.95 [0.30-2.97]; *P* = 0.930, Figure S5A). We then pooled the overall survival and found that anti-PD-1/PD-L1 monotherapy reduced the risk of death in patients with PD-L1 expression ≥1% (HR 0.66 [0.57-0.76]; *P* < 0.001, Figure 3(a)) but not for those with PD-L1 expression <1% (HR 0.87 [0.74-1.03]; *P* = 0.098, Figure 3(a)). By setting 5% or 10% as the cut-off point for positive PD-L1 expression, we noted that anti-PD-1/PD-L1 monotherapy could prolong the overall survival of both PD-L1-positive and PD-L1-negative patients (HR for PD-L1 <5% vs. ≥5% [0.76 vs. 0.56], < 10% vs. ≥10% [0.78 vs. 0.59]; all *P* < 0.05, Figure 3(a)). Like NSCLC, anti-PD-1/PD-L1 combination therapy correlated with prolonged overall survival of these patients regardless of PD-L1 expression (HR for PD-L1 <1% vs. ≥1% [0.67 vs. 0.55], <5% vs. ≥5% [0.52 vs. 0.62], < 10% vs. ≥10% [0.58 vs. 0.51]; all *P* < 0.05, Figure 3(b)).

**Figure 4:**
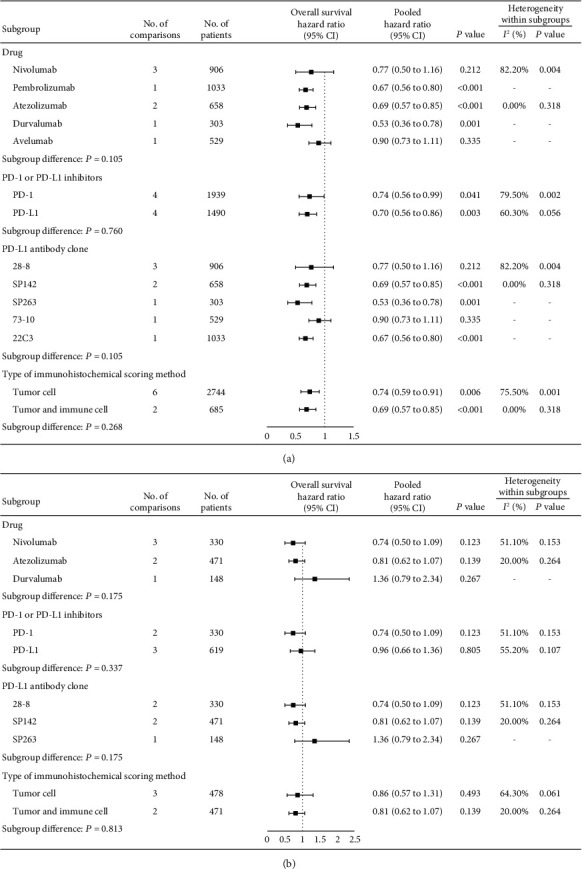
Subgroup analyses for overall survival comparing anti-PD-1/PD-L1 drugs to control treatments in NSCLC patients with PD-L1 expression statuses ≥1% (a) and <1% (b). NSCLC: nonsmall cell lung cancer; PD-1: programmed death 1; PD-L1: programmed death-ligand 1.

**Figure 5:**
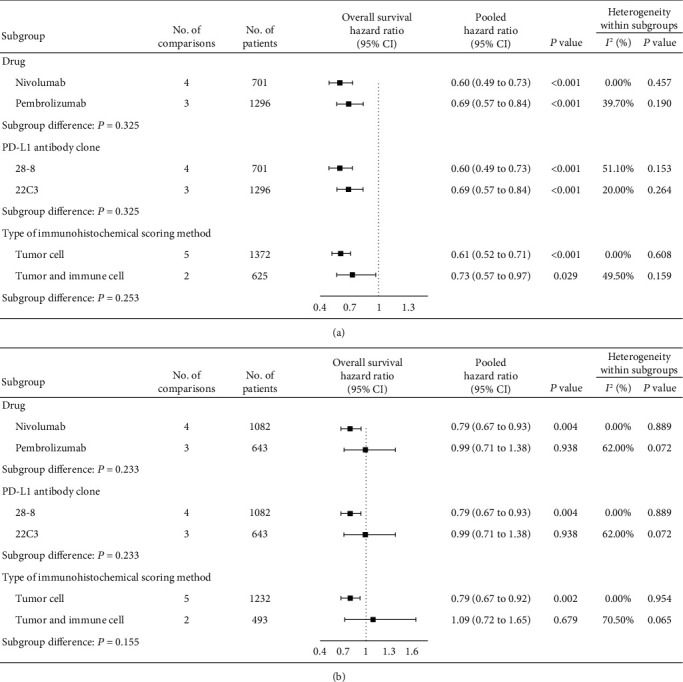
Subgroup analyses for overall survival comparing anti-PD-1/PD-L1 drugs to control treatments in patients with other cancer types with PD-L1 expression statuses ≥1% (a) and <1% (b). PD-1: programmed death 1; PD-L1: programmed death-ligand 1.

In summary, by setting 1% as the cut-off for positive PD-L1 expression, we noted that anti-PD-1/PD-L1 monotherapies increased the tumor response and prolonged the overall survival in patients who were PD-L1 positive but not in those who were PD-L1 negative, compared with control treatments, indicating that 1% may be the suitable cut-off value for patient selection. Therefore, we further performed subgroup analyses to experimental agents, PD-1/PD-L1 inhibitors, PD-L1 antibody clone, and type of IHC scoring method that may affect outcomes. Overall, for these subgroups, patients that were PD-L1 positive (Figure 4(a), Figure 5(a)) rather than PD-L1 negative (Figure 4(b), Figure 5(b)) could benefit from anti-PD-1/PD-L1 monotherapies.

## 4. Discussion

PD-1/PD-L1 inhibitors rely heavily on the tumor microenvironment to work. Theoretically, only patients with inflamed tumors should benefit from immunotherapy; those with other immune types, such as the immune-desert phenotype and immune-excluded tumors, have poor response to immunotherapy due to the absence of immune effector cells in the tumor microenvironment or obstruction between the immune effector cells and tumor cells [45]. Hence, a priority is to identify those patients who could potentially benefit from receiving anti-PD-1/PD-L1 immunotherapy. To address this urgent need, we relied on the data from 24 high-quality, randomized controlled trials that comprised 14,860 subjects and performed pooled analysis to assess the predictive value of PD-L1 expression for anti-PD-1/PD-L1 immunotherapy under different positive expression thresholds. Our findings suggest that patients with different PD-L1 expression levels have different outcomes for anti-PD-1/PD-L1 monotherapies. Overall, we noted that anti-PD-1/PD-L1 monotherapies prolonged the overall survival of patients with PD-L1 expression ≥1%, rather than those with PD-L1 expression <1%, indicating that the therapeutic efficacy of anti-PD-1/PD-L1 monotherapies relied on the expression of PD-L1, and eligible patients were required to have PD-L1 expression levels of at least 1%. Nonetheless, when anti-PD-1/PD-L1 inhibitors were combined with other therapies, favourable outcomes were observed in patients without the restriction of PD-L1 expression.

Among the emerging immune-relevant biomarkers for anti-PD-1/PD-L1 immunotherapy, the expression of PD-L1 on tumor and immune cells is one of the most plausible biomarkers, since it represents the extent of T cell infiltration in the tumor microenvironment [46–48]. Several studies have reported an association between the efficacy of anti-PD-1/PD-L1 therapy and PD-L1 expression [9, 47]. Taking advanced NSCLC as an example, anti-PD-1/PD-L1 monotherapy can improve the tumor response and overall survival of patients with higher PD-L1 expression (KEYNOTE-024 study) [30], but not patients with low PD-L1 expression (CheckMate 026) [32], when compared with platinum-based chemotherapy. Similarly, Kim and colleagues reported that the objective response rate of PD-L1 (+) gastric cancer was significantly higher than that of PD-L1 (-) gastric cancer (50.0% versus 0.0%, *P* < 0.001) [9]. Despite these favourable results, some issues still remain, such as the heterogeneity between the primary tumors and metastatic sites and the dynamic changes of the tumor environment [49]. Hence, it is still debatable whether the PD-L1 expression of archival biopsies at specific time points is predictive during the long-term treatment process; even if it is, the suitable cut-off point remains unknown. A suitable cut-off value of PD-L1 expression should be set based on the conditions that PD-L1-positive, but not PD-L1-negative, patients could benefit from PD-1/PD-L1 inhibitors and that the PD-L1-positive population should be as large as possible. To address these urgent issues, we performed pooled analyses based on 24 high-quality randomized controlled trials with over 14,860 subjects and performed subgroup analyses with respect to different cancer types to verify the robustness of our findings. In summary, our findings demonstrated that patients with different PD-L1 expression levels are associated with discrepant outcomes for anti-PD-1/PD-L1 monotherapies. When we defined PD-L1 ≥1% as positive expression, patients who were PD-L1-positive pretreatment benefitted from the anti-PD-1/PD-L1 monotherapies, indicating that PD-L1 expression is an effective predictive biomarker of anti-PD1/PD-L1 monotherapy response and that 1% may be the suitable cut-off value.

Considering the low response rate of immunotherapy, oncology researchers are now investigating anti-PD1/PD-L1 combination therapies, such as anti-PD-1/PD-L1 agents in combination with chemotherapy [36, 42–44], ipilimumab [29, 38], or bevacizumab [10], for the treatment of cancers. However, while the anti-PD-1/PD-L1 combination therapies prolonged the survival of more but not all treated patients, and these combination therapies increased the number of immune-related adverse events [36, 50]; hence, it is still urgently needed to seek effective biomarkers to distinguish the responders from nonresponders before initial combination therapies. In the present study, we explored the validity of PD-L1 expression as a predictor for anti-PD-1/PD-L1 combination therapies and found that anti-PD-1/PD-L1 combination therapies can prolong the overall survival in patients with either high or low PD-L1 expression, indicating that anti-PD-1/PD-L1 combination therapy is effective regardless of pretreatment PD-L1 expression. Thus, PD-L1 expression cannot be recommended as a prognostic biomarker to select patients to receive anti-PD-1/PD-L1 combination therapy. It is known that the interaction between cancer and the immune system is a dynamic process [51]; the expression of PD-L1 can be regulated at the transcriptional, posttranscriptional, and protein levels [7], and the combination of other therapies may promote the activation of dendritic cells, the infiltration of T cells, and the exposure of neoantigens, which result in increases in PD-L1 expression and facilitate the efficacy of the PD-1/PD-L1 blockade. This is one possible reason why patients with little or no PD-L1 expression in baseline tumor biopsies can still benefit from anti-PD-1/PD-L1 combination therapies.

Our results have several clinical and research implications. Foremost, while the PD-1/PD-L1 inhibitors have favourable effects on survival, it also comes with trade-offs in immune-related side effects [15]. The average wholesale price for one dose of anti-PD-1/PD-L1 agent has been as high as $5732, which creates tremendous medical costs for patients, families, and the National Health Service annually [52]. Our results showed that PD-L1 expression is a predictor of patient survival and patient response to anti-PD-1/PD-L1 monotherapy and that 1% may be the suitable cut-off value. These findings may facilitate the administration of anti-PD-1/PD-L1 agents, avoid unnecessary immunotherapy for cancer patients, and assist in recruiting eligible patients for future clinical trials. Additionally, PD-L1 expression is not an adequate predictor for anti-PD-1/PD-L1 combination therapies and thus cannot be the routine marker in clinical practice for patient selection for combination therapies. Finally, our results can spur the exploration of other biomarkers, such as PD-L1 expression on circulating tumor cells or exosomes, and tumor mutation burden to enhance the predictive power for combination therapies.

Our study has several notable limitations. First, we observed obvious heterogeneity among the eligible studies. We believe that the heterogeneity was mainly due to the multiple cancer types and multiple-line therapies used in these trials. Hence, we used a random effects model in this study and performed subgroup analyses stratified by different cancer types and treatment strategies to minimize the heterogeneity and verify the robustness of our results. Moreover, no publication bias was observed in this study. Our findings confirmed the correlations between PD-L1 expression and the efficacy of anti-PD-1/PD-L1 immunotherapy. Second, not all the included trials reported PD-L1 expression cut-offs of 1%, 5%, 10%, and 50%; thus, the pooled results in several subgroup analyses were conducted through limited trials. Third, our study only focused on one factor: PD-L1 expression. We recognized that other clinical parameters that may affect outcomes. Thus, we also performed subgroup analyses to experimental agents, PD-1/PD-L1 inhibitors, PD-L1 antibody clone, and type of IHC scoring method to further validate the robustness of our findings. Fourth, this study was not designed based on an a priori protocol, indicating the potentially biased methods. Finally, our study was carried out at the trial level instead of at the individual level. Therefore, we failed to test the correlations between PD-L1 expression and efficacy in specific subgroups according to clinicopathological characteristics.

## 5. Conclusions

Compared with conventional therapy, anti-PD-1/PD-L1 monotherapies prolonged the overall survival of patients with PD-L1 expression ≥1%, but not those with PD-L1 expression <1%, regardless of cancer type; anti-PD-1/PD-L1 combination therapies reduced the risk of death in different cancer types, regardless of PD-L1 expression. Therefore, we recommend PD-L1 expression as a predictive biomarker in patient selection for anti-PD-1/PD-L1 monotherapies, but not for combination therapies. In the future, researchers should consider the impact of PD-L1 expression to better design the anti-PD1/PD-L1 clinical trials.

## Figures and Tables

**Table 1:** Characteristics of the included trials.

Studies	Phase	Population	Treatment	PD-L1 antibody clone	IHC scoring method	Cutoff for PD-L1 positivity	Patients number	Number of the patients by PD-L1 expression status		Follow-up (months)		Studies
								Experimental arm		Control arm		
								PD-L1 positive	PD-L1 negative	PD-L1 positive	PD-L1 negative	
Motzer (2015) [29]	3	RCC	Nivolumab vs. everolimus	28-8	Type 1	≥1%	821	276	94	299	87	NR
Robert (2015) [30]	3	Melanoma	Nivolumab vs. dacarbazine	28-8	Type 1	≥5%	418	74	136	74	134	16.7
Borghaei (2015) [12]	3	Nonsquamous NSCLC	Nivolumab vs. docetaxel	28-8	Type 1	≥1%	582	108	123	101	123	13.2
Brahmer (2015) [28]	3	Squamous-cell NSCLC	Nivolumab vs. docetaxel	28-8	Type 1	≥1%	272	54	63	52	56	11
Fehrenbacher (2016) [13]	2	NSCLC	Atezolizumab vs. docetaxel	SP142	Type 2	≥1%	287	93	51	102	41	13.0
Reck (2016) [33]	3	NSCLC	Pembrolizumab vs. chemotherapy	22C3	Type 1	≥50%^∗^	305	154		151		11.2
Ferris (2016) [16]	3	Head and neck cancer	Nivolumab vs. chemotherapy	28-8	Type 1	≥1%	361	88	73	61	38	5.1
Hodi (2016) [32]	2	Melanoma	Nivobumab+ipilimumab vs. ipilimumab	28-8	Type 1	≥5%	142	56	24	27	11	24.5
Herbst (2016) [31]	2/3	NSCLC	Nivolumab^∗∗^ vs. docetaxel	22C3	Type 1	≥1%	1033	690		343		13.1
Schachter (2017) [37]	3	Melanoma	Pembrolizumab^∗∗∗^ vs. ipilimumab	22C3	Type 1	≥1%	834	446	103	225	47	22.9
Rittmeyer (2017) [36]	3	NSCLC	Atezolizumab vs. docetaxel	SP142	Type 2	≥1%	850	241	180	222	199	21
Wolchok (2017) [17]	3	Melanoma	Nivolumab+ipilimumab; nivolumab^∗∗∗∗^ vs. ipilimumab	28-8	Type 1	≥1%	945	68; 80	210; 208	75	202	35.7 vs. 38 vs. 18.6
Larkin (2017) [18]	3	Melanoma	Nivolumab vs. chemotherapy	28-8	Type 1	≥5%	405	134	138	67	66	24
Bellmunt (2017) [19]	3	Urothelial carcinoma	Pembrolizumab vs. chemotherapy	22C3	Type 2	≥10%	542	74	186	90	176	14.1
Carbone (2017) [37]	3	NSCLC	Nivolumab vs. IC chemotherapy C	28-8	Type 1	≥5%	541	208	63	210	60	13.5
Kang (2017) [35]	3	Gastric cancer	Nivolumab vs. placebo	28-8	Type 1	≥1%	493	114	16	52	10	8.8
Gandhi (2018) [38]	3	NSCLC	Chemotherapy+pembrolizumab vs. chemotherapy+placebo	22C3	Type 1	≥1%	616	260	127	128	63	10.5
Shitara (2018) [40]	3	Gastric or gastroesophageal junction cancer	Pembrolizumab vs. paclitaxel	22C3	Type 2	≥1%	592	196	99	199	96	8.5
Motzer (2018) [39]	3	RCC	Nivolumab+ipilimumab vs. sunitinib	28-8	Type 1	≥1%	847	100	284	114	278	25.2
Antonia (2018) [14]	3	NSCLC	Durvalumab after chemodadiotherapy vs. placebo after chemodadiotherapy	SP263	Type 1	≥1%	713	212	90	91	58	25.2
Barlesi (2018) [41]	3	NSCLC	Avelumab vs. docetacel	73-10	Type 1	≥1%	692	264	129	265	132	18.3
Paz-Ares (2018) [42]	3	Squamous NSCLC	Pembrolizumab+chemotherapy vs. placebo+chemotherapy	22C3	Type 1	≥1%	559	176	95	177	99	7.8
Schmid (2018) [43]	3	TNBC	Atezolizumab+nabpaclitaxel vs. placebo+nabpaclitaxel	SP142	Type 2	≥1%	902	185	266	184	267	12.9
Socinski (2018) [44]	3	NSCLC	ABCP^∗∗∗∗∗^ vs. BCP	SP142	Type 2	≥1%	692	209	166	195	172	15.4

NSCLC: nonsmall cell lung cancer; RCC: renal cell carcinoma; TNBC: triple-negative breast cancer; NR: not reached; IHC: immunohistochemistry; Type 1 = membranous staining on tumor cells; Type 2 = membranous or cytoplasmic staining, or both, of tumor cells and tumor-infiltrating immune cells; ^∗^ all the included patients in KEYNOTE-024 were patients with PD-L1 ≥50%; ^∗∗^ the experimental arm of KEYNOTE-010 comprised two cohorts: pembrolizumab 2 mg/kg (*n* = 345) and pembrolizumab 10 mg/kg (*n* = 346), and all the included patients in KEYNOTE-010 were patients with PD-L1 ≥1%; ^∗∗∗^ the experimental arm of KEYNOTE-006 comprised two cohorts: intravenous pembrolizumab every 2 weeks (*n* = 279) and intravenous pembrolizumab every 3 weeks (*n* = 277); here, we combined these two cohorts as the experimental arm to compare with ipilimumab group (control arm; *n* = 278); ^∗∗∗∗^ the experimental arm of CheckMate 067 comprised two cohorts: nivolumab-plus-ipilimumab group (*n* = 314) and nivolumab group (*n* = 316); these two cohorts were compared with ipilimumab group (*n* = 315), respectively; ^∗∗∗∗∗^ the IMpower150 had three cohorts: BCP cohort (bevacizumab plus carboplatin plus paclitaxel), ACP cohort (atezolizumab plus carboplatin plus paclitaxel), and ABCP cohort (atezolizumab plus BCP); here, the ABCP cohort was compared with the BCP cohort before the ACP cohort was compared with the BCP cohort.

## Data Availability

All data used to support the findings of this study are included within the article.

## Supplementary Materials

Supplementary Materials Box 1: PubMed search terms. Table S1: risk of bias of the included trials. Figure S1: study flow diagram of included studies in this systematic review and meta-analysis. A total of 486 related studies after the initial search strategy; 436 studies were further excluded after screening the title and abstract. After review of the full-text of 50 articles, 27 studies were further excluded because of the following reason: noncomparative trial (*n* = 21) [25–45], no report of OS (*n* = 3) [46–48], and no report of OS by PD-L1 expression (*n* = 3) [49–51]. We then performed a careful manual search and identified one study for inclusion. Hence, a total of 24 trials were included for the quantitative synthesis and meta-analysis. Figure S2: Begg's funnel plot for publication bias test (*P* = 0.137). Each circle represents a separate study for indicated association, and horizontal line represents the mean effect size. Figure S3: forest plot of objective response rate in patients treated with anti-PD-1/PD-L1 drugs versus control. Figure S4: forest plot of objective response rate comparing anti-PD-1/PD-L1 drugs to control treatment in NSCLC patients with different PD-L1 expression status. NSCLC: nonsmall cell lung cancer; PD-1: programmed death 1; PD-L1: programmed death-ligand 1. Figure S5: forest plot of objective response rate comparing anti-PD-1/PD-L1 drugs to control treatment in patients with other cancer types with different PD-L1 expression status. PD-1: programmed death 1; PD-L1: programmed death-ligand 1.
