# Supplementary material for: The Value of PD-L1 Expression in Predicting the Efficacy of Anti-PD-1 or Anti-PD-L1 Therapy in Patients with Cancer: A Systematic Review and Meta-Analysis
Source: Dis Markers. 2020 Dec 16;2020:6717912. doi: 10.1155/2020/6717912 (PMC7787798; doi:10.1155/2020/6717912)
Supplement: Supplementary Materials — Box 1: PubMed search terms. Table S1: risk of bias of the included trials. Figure S1: study flow diagram of included studies in this systematic review and meta-analysis. A total of 486 related studies after the initial search strategy; 436 studies were further excluded after screening the title and abstract. After review of the full-text of 50 articles, 27 studies were further excluded because of the following reason: noncomparative trial (n = 21) [25–45], no report of OS (n = 3) [46–48], and no report of OS by PD-L1 expression (n = 3) [49–51]. We then performed a careful manual search and identified one study for inclusion. Hence, a total of 24 trials were included for the quantitative synthesis and meta-analysis. Figure S2: Begg's funnel plot for publication bias test (P = 0.137). Each circle represents a separate study for indicated association, and horizontal line represents the mean effect size. Figure S3: forest plot of objective response rate in patients treated with anti-PD-1/PD-L1 drugs versus control. Figure S4: forest plot of objective response rate comparing anti-PD-1/PD-L1 drugs to control treatment in NSCLC patients with different PD-L1 expression status. NSCLC: nonsmall cell lung cancer; PD-1: programmed death 1; PD-L1: programmed death-ligand 1. Figure S5: forest plot of objective response rate comparing anti-PD-1/PD-L1 drugs to control treatment in patients with other cancer types with different PD-L1 expression status. PD-1: programmed death 1; PD-L1: programmed death-ligand 1. [file 6717912.f1.docx]

**Box 1: PubMed search terms:**

(“nivolumab”[Supplementary Concept] OR “Nivolumab”[tiab] OR “Opdivo”[tiab] OR “MDX-1106”[tiab] OR “ONO-4538”[tiab] OR “BMS-936558”[tiab] OR “NIVO”[tiab] OR “pembrolizumab”[Supplementary Concept] OR “pembrolizumab”[tiab] OR “lambrolizumab”[tiab] OR “keytruda”[tiab] OR “MK-3475”[tiab] OR “SCH 900475”[tiab] OR “avelumab”[Supplementary Concept] OR “Avelumab”[tiab] OR “MSB0010718C”[tiab] OR “MPDL3280A”[Supplementary Concept] OR “MPDL3280A”[tiab] OR “atezolizumab”[tiab] OR “Tecentriq”[tiab] OR “RG7446”[tiab] OR “RO5541267”[tiab] OR “Durvalumab”[tiab] OR “MEDI4736”[tiab] OR “MEDI-4736”[tiab] OR checkpoint inhibitor*[tiab] OR “PD-1”[tiab] OR “PDL1”[tiab]) AND (Clinical Trial, Phase III[ptyp] OR “phase 3 clinical trial”[tiab] OR “phase III clinical trial”[tiab] OR “phase 3 trial”[tiab] OR “phase III trial”[tiab] OR “phase 3 clinical study”[tiab] OR “phase III clinical study”[tiab] OR “phase 3 study”[tiab] OR “phase III study”[tiab] OR “phase 3 randomized trial”[tiab] OR “phase III randomized trial”[tiab] OR Clinical Trial, Phase II[ptyp] OR “phase 2 clinical trial”[tiab] OR “phase II clinical trial”[tiab] OR “phase 2 trial”[tiab] OR “phase II trial”[tiab] OR “phase 2 clinical study”[tiab] OR “phase II clinical study”[tiab] OR “phase 2 randomized trial”[tiab] OR “phase II randomized  trial”[tiab] OR “phase 2 study”[tiab] OR “phase II study”[tiab] OR “phase 2/3 clinical trial”[tiab] OR “phase II/III clinical trial”[tiab] OR “phase 2/3 trial”[tiab] OR “phase II/III trial”[tiab] OR “phase 2/3 clinical study”[tiab] OR “phase II/ III clinical study”[tiab] OR “phase 2/3 study”[tiab] OR “phase II/III study”[tiab] OR “phase 2/3 randomized trial”[tiab] OR “phase II/III randomized trial”[tiab] OR Randomized  Controlled Trial[ptyp] OR “randomized controlled trial”[tiab] OR “RCT”[tiab])

**Table S1. Risk of bias of the included trials**

| **Study** | **Study number** | **Randomization** | **Allocation concealment** | **Blinding of participants**  **and staﬀ** | **Blinding of outcome**  **assessors** | **Incomplete outcome data addressed** | **Selective outcomes reporting** | **Other bias** |
| --- | --- | --- | --- | --- | --- | --- | --- | --- |
| Motzer (2015)[1] | CheckMate 025 | Low | Low | High | Low | High | High | Low |
| Robert (2015)[2] | CheckMate 066 | Low | Low | Low | Low | High | High | Low |
| Borghaei (2015)[3] | CheckMate 057 | Low | Low | High | High | High | High | Low |
| Brahmer (2015)[4] | CheckMate 017 | Low | Low | High | High | High | High | Low |
| Ferris (2016)[5] | CheckMate 141 | Low | Low | High | High | High | High | Low |
| Hodi (2016)[6] | CheckMate 069 | Low | Low | High | High | High | High | Low |
| Herbst (2016)[7] | KEYNOTE-10 | Low | Low | High | High | High | High | Low |
| Fehrenbacher (2016)[8] | POPLAR | Low | Low | High | High | High | High | Low |
| Reck (2016)[9] | KEYNOTE-024 | Low | High | High | High | High | Low | Low |
| Bellmunt (2017)[10] | KEYNOTE-045 | Low | Unclear | High | High | High | High | Low |
| Carbone (2017)[11] | CheckMate 026 | Low | Low | High | High | High | High | Low |
| Kang (2017)[12] | ATTRACTION-2 | Low | Low | Low | Low | High | High | Low |
| Rittmeyer (2017)[13] | OAK | Low | Low | High | High | High | High | Low |
| Schachter (2017)[14] | KEYNOTE-006 | Low | Low | High | High | High | High | Low |
| Wolchok (2017)[15] | CheckMate 067 | Low | Low | Low | Low | Low | Low | Low |
| Gandhi (2018)[16] | KEYNOTE -189 | Low | Low | Low | Low | High | Low | Low |
| Larkin (2017)[17] | CheckMate 037 | Low | Unclear | High | High | High | High | Low |
| Motzer (2018)[18] | CheckMate 214 | Low | Low | High | High | High | Low | Low |
| Shitara (2018)[19] | KEYNOTE-061 | Low | Low | Low | Low | High | High | Low |
| Antonia (2018) [20] | PACIFIC | Low | Low | Low | Low | Low | High | Low |
| Barlesi (2018) [21] | JAVELIN Lung 200 | Low | Low | Low | Unclear | High | High | Low |
| Paz-Ares (2018) [22] | KEYNOTE-407 | Low | Low | Low | Low | Low | High | Low |
| Schmid (2018) [23] | IMpassion130 | Low | Low | Low | Low | High | High | Low |
| Socinski (2018) [24] | IMpower150 | Low | Low | High | High | High | High | Low |


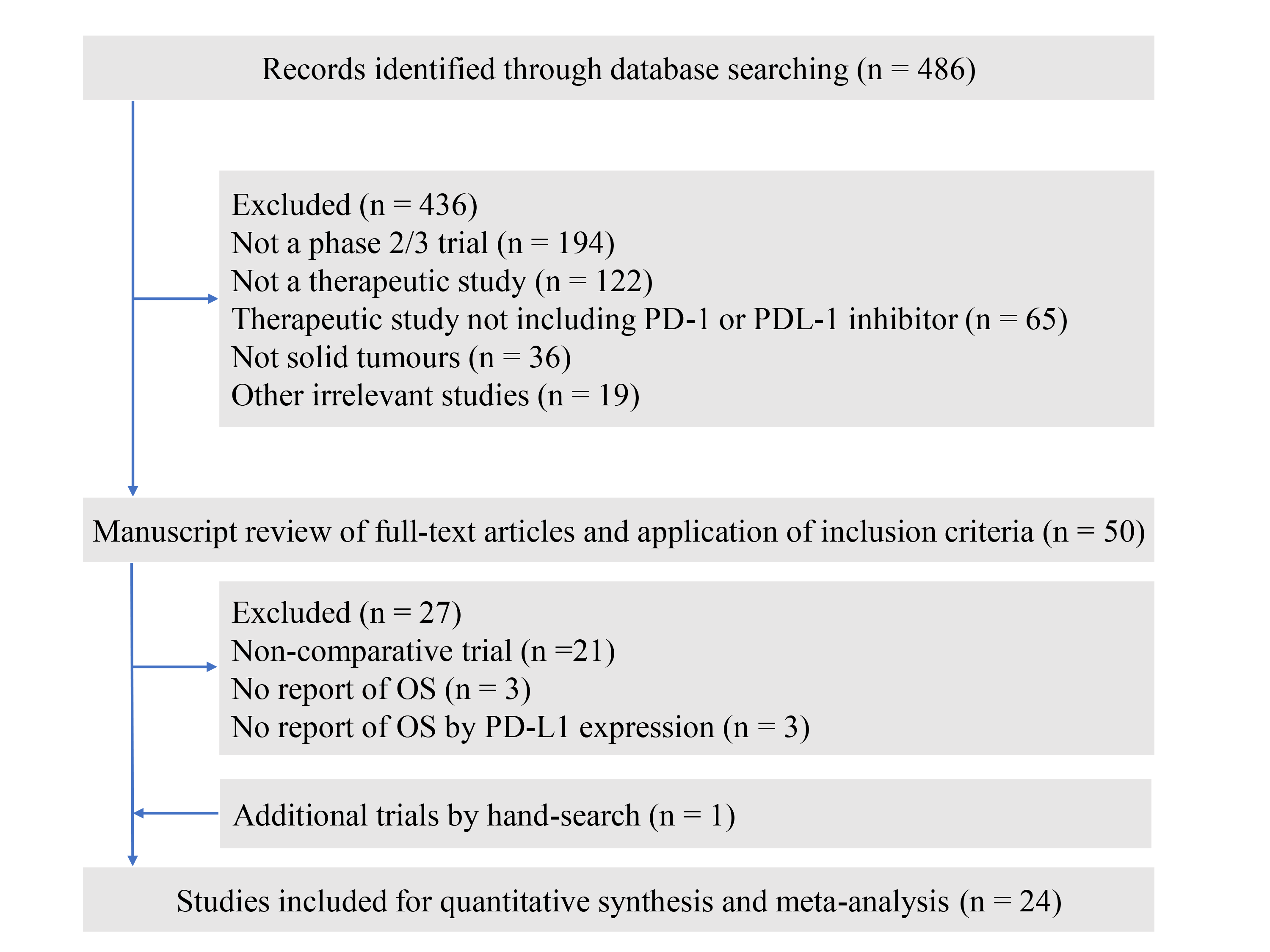


**Figure S1.** Study flow diagram of included studies in this systematic review and meta-analysis. A total of 486 related studies after the initial search strategy; 436 studies were further excluded after screening the title and abstract. After review of the full-text of 50 articles, 27 studies were further excluded because of the following reason: non-comparative trial (n=21) [25-45], no report of OS (n=3) [46-48]and no report of OS by PD-L1 expression (n=3) [49-51]. We then performed a careful manual search and identified one study for inclusion. Hence, a total of 24 trials were included for the quantitative synthesis and meta-analysis


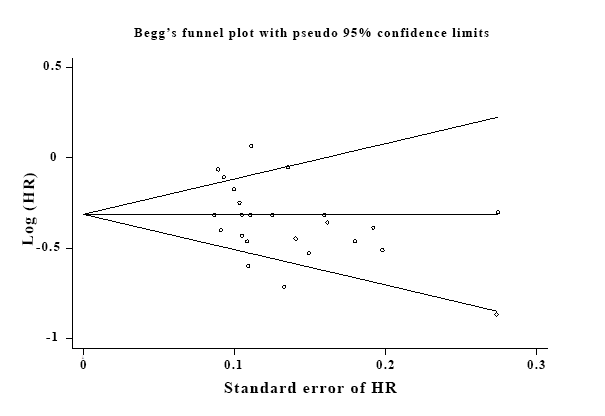


**Figure S2.** Begg’s funnel plot for publication bias test (P = 0.137). Each circle represents a separate study for indicated association, and horizontal line represents the mean effect size.


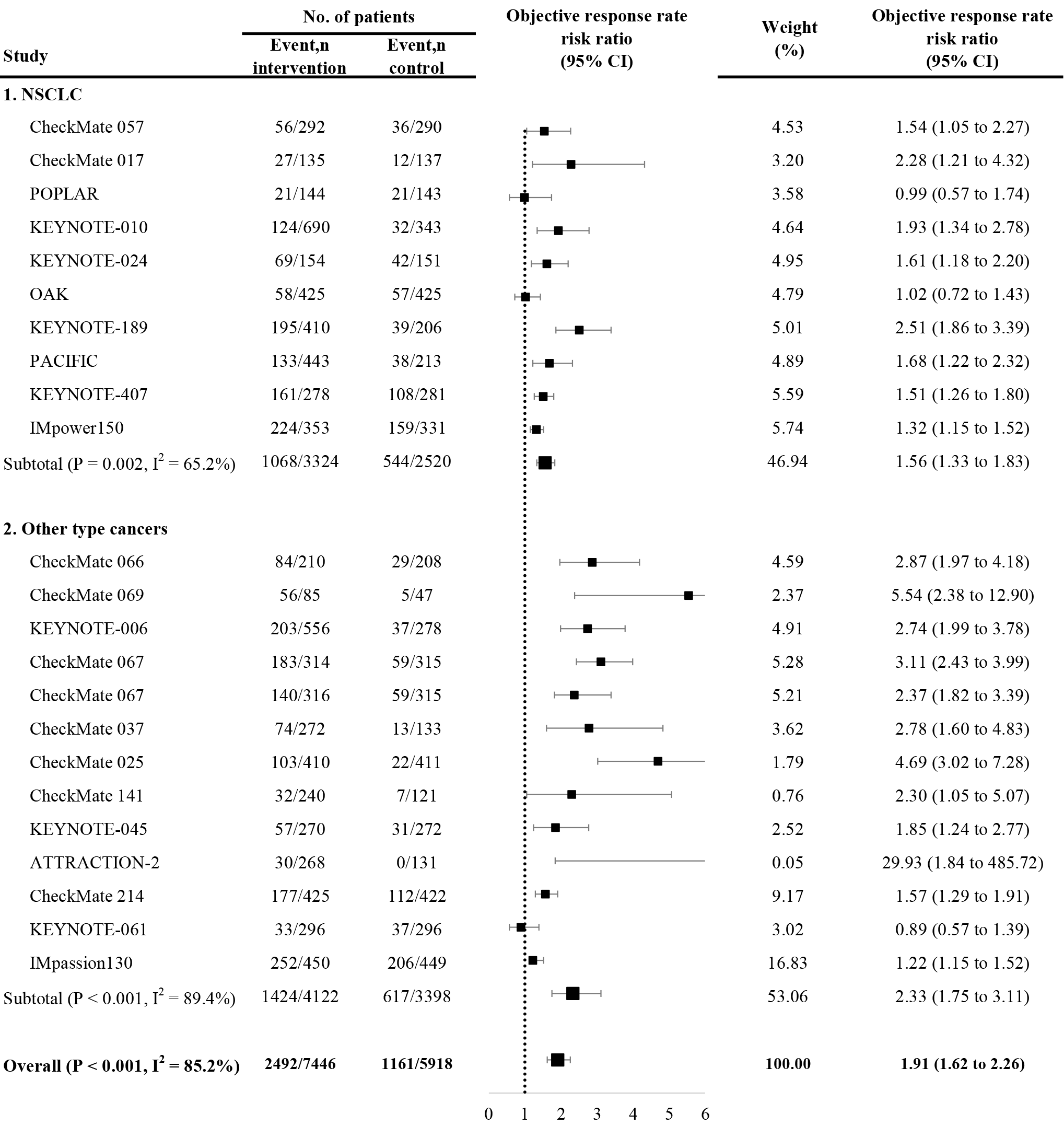


**Figure S3.** Forest plot of objective response rate in patients treated with anti-PD-1/PD-L1 drugs versus control.


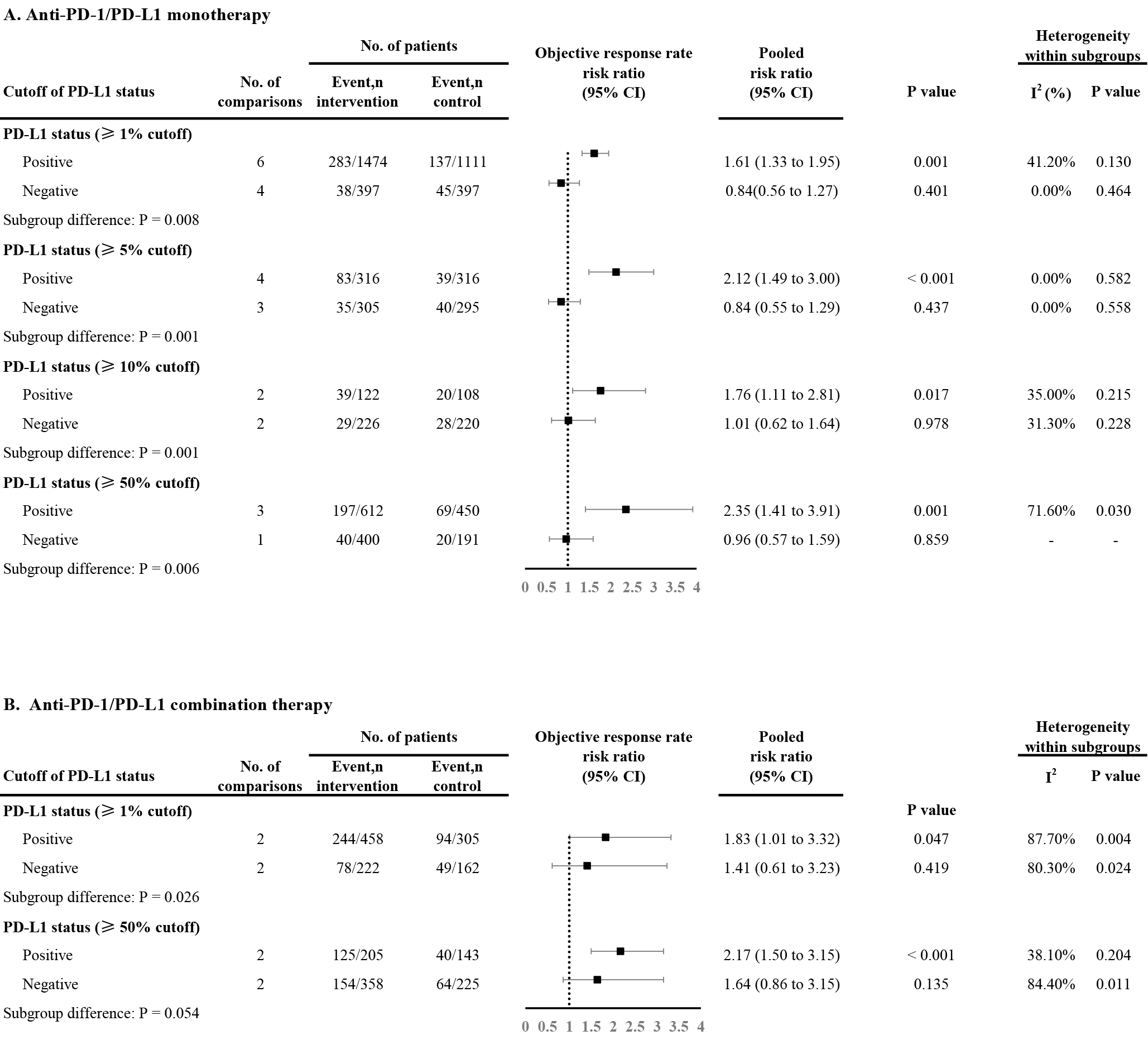


**Figure S4.** Forest plot of objective response rate comparing anti-PD-1/PD-L1 drugs to control treatment in NSCLC patients with different PD-L1 expression status. NSCLC, non-small cell lung cancer; PD-1, programmed death 1; PD-L1, programmed death-ligand 1


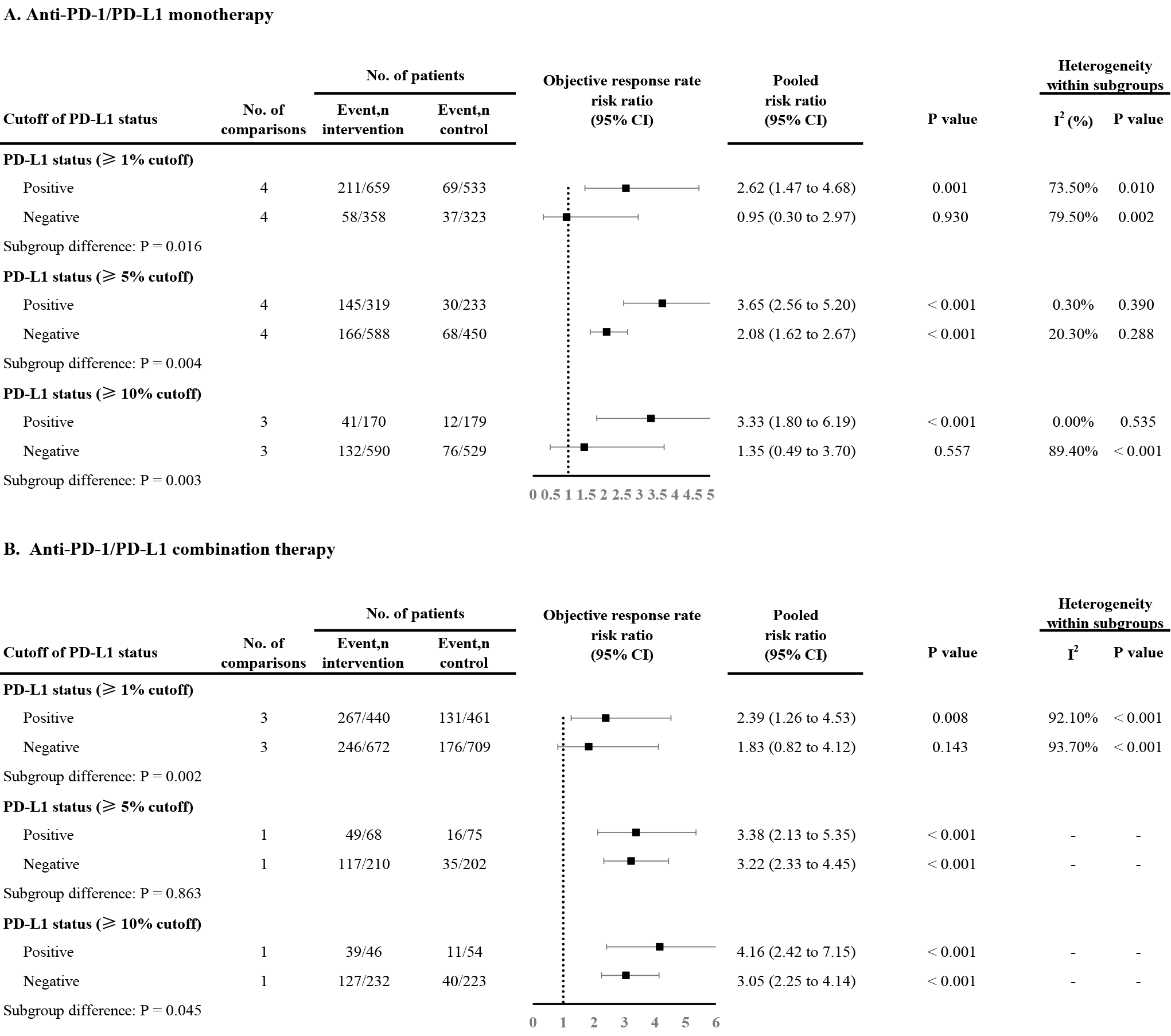


**Figure S5.** Forest plot of objective response rate comparing anti-PD-1/PD-L1 drugs to control treatment in patients with other cancer types with different PD-L1 expression status. PD-1, programmed death 1; PD-L1, programmed death-ligand 1
